# Towards Social Capital in a Network Organization: A Conceptual Model and an Empirical Approach

**DOI:** 10.3390/e22050519

**Published:** 2020-05-01

**Authors:** Saad Alqithami, Rahmat Budiarto, Musaad Alzahrani, Henry Hexmoor

**Affiliations:** 1 Department of Computer Science, Albaha University, Al Bahah 65527, Saudi Arabia; Rahmat@bu.edu.sa (R.B.); Malzahr@bu.edu.sa (M.A.); 2 Department of Computer Science, Southern Illinois University, Carbondale, IL 62901, USA; Hexmoor@cs.siu.edu

**Keywords:** social capital, open multi-agent systems, collaboration, interaction, complex networks

## Abstract

Due to the complexity of an open multi-agent system, agents’ interactions are instantiated spontaneously, resulting in beneficent collaborations with one another for mutual actions that are beyond one’s current capabilities. Repeated patterns of interactions shape a feature of their organizational structure when those agents self-organize themselves for a long-term objective. This paper, therefore, aims to provide an understanding of social capital in organizations that are open membership multi-agent systems with an emphasis in our formulation on the dynamic network of social interactions that, in part, elucidate evolving structures and impromptu topologies of networks. We model an open source project as an organizational network and provide definitions and formulations to correlate the proposed mechanism of social capital with the achievement of an organizational charter, for example, optimized productivity. To empirically evaluate our model, we conducted a case study of an open source software project to demonstrate how social capital can be created and measured within this type of organization. The results indicate that the values of social capital are positively proportional towards optimizing agents’ productivity into successful completion of the project.

## 1. Introduction

There has been an increasing interest in service-oriented computing that aims to combine computational resources dynamically across boundaries, e.g., semantic web and peer-to-peer networking. A shared feature of all these systems is that different services from data and software can be invoked remotely to achieve a common goal, i.e., organization oriented. This style of distributed services may often allow a number of competing service providers to achieve their respective requirements due to the applicability of shared resources. Nevertheless, the collaborative nature of these systems means that they will invariably create uncertainty surrounding the incentives of agents offering these services. Multi-agent systems (MAS), in this regard, have demonstrated their efficiency in modeling and implementing distributed systems as it signifies dynamics of heterogeneous agents’ interactions.

An ad-hoc organization of networked agents may form to rally around a specific problem. We explore the effects resultant from networking by addressing one type of network effects called *Social Capital* (SC). Social capital in a cross-organizational network can be characterized as collocated or virtual collaboration to produce successful outcomes and successful connections. There are two major perspectives on SC in networks. In the macroscopic perspective, SC for the entire network is considered. In this view individuals do not incrementally add to the system or withdraw units of SC. Instead, the foci are on the system principles like norms and conventions that provide resources for overall social welfare. In contrast, the microscopic perspective adopted here explores how individuals can gain access to resources by their positions and connections in the network [1].

### 1.1. Understanding SC

The consideration of SC may transcend issues surrounding the heterogeneity of an agent’s affiliations since it captures benefits resulting from the preferential treatment and collaboration among agents [2]. The scale of SC should consider components of norm, trust, reciprocity, governance, tolerance, friendships and acceptance, which might be bounded within the organizational networks. Quantities of SC can be used to replace interpersonal trust among agents and that is due to when an organization generates positive values of SC, constituent agents gain benevolence and behave in a trusting manner [3]. Other benefits of SC are enhanced group communication, efficient use of intellectual capital, better collective action and easy way of accessing resources [4].

There is still no theoretical or practical value in determining some quantifiable measures of SC, and the main idea is of the driven value of SC for an agent or an organization. Han and Breiger [5] are of the ones to propose a measurement of SC. There are a few main elements of the SC that have a proportional relationship to one another. Topologically speaking, high bonding rates provide more opportunities for interaction and growth of SC. However, network structure by itself is inadequate for the determination of SC. We must examine the contents of interaction and dispositions that create social forces that attract or repel individuals [6]. At the level of a single link, the nature of social flow, i.e., information flow, in the link leads to accumulation of SC. Social flows can be benevolent and positive or negative and lack benevolence. Whereas positive flow leads to network positive gain in SC, negative flow leads to loss of SC. Apart from social flow, dyadic ties may harbor trust or promote distrust [7]. Trust supports SC whereas distrust erodes it. If the topic of interactions between a pair of agents is centered on the main problem for an organization, that link positively contributes to SC. Thus, flow, trust, and topic are link attributes that are proportional determinants for SC.

Social capital in a *link* is the accumulation of positive values of social flow and trust plus abundance of communication over a common topic. Since considering a topic of interaction is included in the determination of SC over the link, we note that this formulation of social capital is relative only to links in an organization. SC is generated in the links through dynamics of interaction on the links. Thus, SC for a *network* linearly scales by summing SC for all links in the network. Increased values of links are proportional to increase social capital, i.e., network bonding measure. The effects of organization topology are overlooked in this network perspective but will be considered egocentrically. All bridged communities contribute to accumulation of the overall SC, which is the instrumental purpose of SC [8]. From an egocentric perspective, bridging is said to contribute to social capital [3,9].

Network bonding leads to increased density and closure in the network, which increases resource access [1]. The more interconnections a network has, the more opportunities it will have for accumulation of SC. A network with the most links possible, i.e., a clique, supports the highest SC because a clique structure is a complete graph that connects each agent with everyone else in the graph which produces a high bonding rate [10]. Thus, SC is seen in this structure to arrive to a higher value compared with other types of structures. Other organizational structures, by contradiction, yield lower SC than a complete network graph due to their less of connections. Social capital for an *agent*, on a differnt perspective, is the egocentric for an individual that deliberately mirrors the Bonacich Power Index [11]. This coincidence helps us to exploit the topological position of nodes. An agent that is well positioned by having a High Power Index, i.e., high Bonacich centrality value [11], will similarly possess high SC [2].

The previous definitions and views of SC in a restrictive network open the discussion to consider them in organizations. Organizations, in general, are bounded networks with purposeful interaction between their agents. Organizations have multiple degrees of institutionalized culture, norms and values that are essential in the development of SC. SC receives direct and indirect effects from formal institutions due to formal relations that have been provided by the organization to create interpersonal relations that contribute positively to SC [12]. SC in an organization represents the resultant outcome from SCs embedded in a social system or through a direct or indirect social relations of an agent, including inherited norms and culture values. Values of SC are built within the social structure to facilitate the agents’ actions and interaction [12].

### 1.2. SC in the Literature

Social capital has been studied by many previous researchers [13,14,15,16,17,18,19,20]; however, a unified definition of it is a critical issue. Bourdieu [21] refers to SC as the actual or potential collective resources in an institutionalized synergistic network of homogenous agents, which in some cases may result into other forms of capitals. The point behind SC is to make use of the accumulation of resources embedded in the social structure [22]. Other authors [23] have defined SC as an attribute of individuals that enhances their abilities to solve collective action problems. Furthermore, Nahapiet and Ghoshal [14] described SC through three different dimensions: (1) *structure dimension* to include the properties of the whole network, (2) *relational dimension* to present the values of exchanges in agents’ connections, and (3) *cognitive dimension* to support the homogeneity by sharing interpretations and mutual understanding between agents [14,16].

There are two types of social capital in an intra-organizational network: bonding and bridging [24]. Both types are generated from agents’ interaction, i.e., network homophily—the theory of homophily helps in initiating attachments or interactions between agents with similar attributes, which allows them to self-select based on their public profiles [25]. A major difference between those two types is that bonding SC occurs between homogenous agents working on a common goal while bridging involves interaction between heterogeneous agents who are not necessarily working for the same goal [22]. Bonding SC increases through closure, which contributes positively to the values of relations. Although bridging SC can be considered between agents within an organization, increase of its value can, in some cases, be a resultant of interaction through an inter-organizational network and bridging cross structural holes [26,27]. Social network analysis, presented in [28], studied a network of wildlife tourism micro-entrepreneurs for the purpose of identifying forms of bonding and bridging social capital. The results showed that interactions, e.g., customer exchange or referral, between the micro-entrepreneurs fostered the formation of a bridging network structure that contains four ties connecting potential sub-groups in the network. In addition, the results highlight the importance of reciprocation between the micro-entrepreneurs for the success of the wildlife tourism business.

From an empirical perspective, Zou, et al. [29] conducted an experiment to investigate the relationships between social capital, emotion experience and life satisfaction for sustainable community. The results revealed that structural social capital and cognitive social capital of the community positively influence the life satisfaction and joyous experience of the residents. However, they have negative impact on painful experience of the residents. Moreover, Sung-Hoon et al. [30] empirically explored the effects of social capital on Organizational Citizenship Behavior (OCB) in the emotional labor context. The empirical study involved 330 participants from South Korea occupying customer service-oriented positions. The results showed that there is a statistically significant positive relationship between social capital and OCB that is sequentially mediated by deep acting and job engagement. The impact of social capital and psychological capital on the entrepreneurial performance of the new generation of migrant workers in China was quantitively analyzed in [31]. Quantitative data were collected through a survey conducted on 525 rural households. The collected data was analyzed using the structural equation modeling. The analysis of data showed that psychological capital and social capital of the new generation of migrant workers have impact on their entrepreneurial outcomes.

The rich literature is a good addition to our view of SC, yet it falls short in differentiating SC from social network analysis that is directly affected by the network topologies. Analyses on the structural dimension, e.g., asymmetric emerging distribution of interrelations, of social capital considering the impact of it on the success of *Open Source Software* (OSS) projects have been discussed in [4,32]. Open source refers to any program in which the source code is made available for use or modification as users or other developers see fit. Open source software is usually developed as a public collaboration and made freely available. The deployment of valuable parameters discussed in the literature into this work in order to efficiently measure and exploit SC in an OSS results in several benefits. One of the reasons for this is that traditional studies on SC consider only the total number of ties an individual or organization has, ignoring the direction of the social flows. In our measurements, however, we signify the inputs by considering the reciprocity exchange theory to measure it. In addition, we consider the impact of SC on a real-world case study of an OSS project, which has set it apart from traditional prior techniques. We take advantage of GitHub (https://github.com/) since it is the most popular platform for open source collaboration. On GitHub, developers can join and contribute to projects by submitting issues and contributing code. They submit issues when sending messages about errors in applications and suggesting ways to fix them. The contribution of code involves sending pull requests with the corrections and improvements. A project team is considered as an organizational network, which consists of developers as nodes and each one may have relations with others through common tasks in modules.

To this end, we have introduced SC and briefly provided some related literature review, i.e., in Section 1. The rest of the article is organized as follows: In Section 2, we quantify social capital from the ground-up starting from agents’ interactions to providing a measurement for the value of SC for an agent and the organization. Section 3 contributes an extensive experiment on an open source project development and details a discussion, while Section 4 draws conclusions and future possibilities.

## 2. Quantifying Social Capital

We consider SC to be a scalar value that can be accumulated as well as consumed either verbatim or used as credit. In a network, SC might be used to trade for help or exchanges with others in the form of delegation of tasks. Bartering with SC can be limited to a pair of agents through an immediate link between them. Alternatively, an agent might enter bartering anonymously with another agent with whom there may not be a directed relationship. Our measurements of SC on OSS Project is based on a weighted task-based directed graph inherited from the general dependency-network graph. An organization, i.e., the OSS Project in our case, is modeled as a directed graph of agents that are contributors as vertices and their cumulative values of relations between the contributors as edges: $\left\{N,R_{elation}\right\}$, where N is the set of agents in an organization that is ≥2, and $R_{elation}\subseteq N\times N$ is the set of directed relations between agents. The organization has a common goal that is divided into a set of tasks. Each task will be conducted by a subgroup of N⊆N. Each agent has a capacity extracted from her public profiles, which include capability, willingness and previous relations.

### 2.1. Parameters of SC

We propose a measurement of relations from continual interaction and a quantification of an agent’s capacity before attempting to measure the values of SC.

#### 2.1.1. Relations Measure

In a dynamic organization, agents form subgroups when they tackle different problems for the continuation of their organization. Even though their relations have a huge impact on the formation as well as the coordination in this world, subgroup formation as well as task or problem allocation is outside the scope of this work. We focus on measuring a network of relationships for subsequent determinations of different values that an agent accumulates when interacting with others. The initial values of relations are provided by every agent when she first joins an organization. Those relations and their values are not static and agents are able to create, diminish, or improve each one of them depending on current actions and interaction.

For every action an organization performs, there exists a goal $G_{j}\in \left\{G\right\}$. Each goal will be distributed into a set of tasks, such that $G_{j}\to \left\{\Theta \right\}=\langle \theta_{1},\cdots ,\theta_{n}\rangle$, for possible assignments to agents. The completion of one task $\theta_{m}\in \left\{\Theta \right\}$ includes interaction between agents for a set of subtasks $\left\{\theta_{m}\right\}=\langle \theta_{m}^{1},\cdots ,\theta_{m}^{k}\rangle$. The coordination as well as control of those tasks are determined by the organization. We benefit from the dynamic interaction among agents while achieving multiple tasks in order to update the current values of relations. Those values of relations depends on the nature of interaction over every given task; therefore, we model relations in a task-based scenario to describe the continual changes over time in inter-agent connections and to help with updating relations throughout repeated task assignments. In the case of OSS Project, the goal is to develop the project and the sub-goals are the releases of the project. The tasks are the software modules that need to be developed in order to achieve a sub-goal, i.e., releasing the project. The subtasks of one module are the lines of codes to be added or deleted in order to complete the module. The interaction between agents (contributors) working in a task (module) occur through the completion of the subtasks.

For every self-selected task, agents define a task-based graph upon the initial relations and there is at least one active edge that prescribes a plan. Agents are able to form an edge through successive interaction. In other words, the network structure allows for the property of transitivity, which permits interaction over that edge to improve giving it the chance to reach a threshold in order to be considered active. Interactions are commonly observed of two types of affinities [3], where (a) *explicit affinities* become evident through interaction over an existing relation, i.e., it is observed when two or more agents have interaction with whom they have a previous experience over an existing edge in the graph, and (b) *implicit affinities* allow for other possible interaction among agents without previously modeled relations. In the case of OSS Project, explicit affinities between two agents exist when both agents contributed on a common software module. Interactions emerge from the closure property of relations [33] and may help in forming new edges when updating relations, i.e., previously un-modeled relations.

The current values of relations are updated every time interval Δt and, in our case, the time interval *t* is the time between releases. For the general assembly, we describe existing relations as explicit links; otherwise, they will be considered as implicit. Values of links are proportional to the frequency of interaction over them. The value on an explicit edge, $EL_{ink}$, between agent $i,i^{\prime }\in N$, is computed accumulatively based on the frequency of interaction, i.e., *I*, between the two agents throughout the time interval, i.e., $\Delta t=t_{2}-t_{1}$. This is stated in Equation (1) at a specific subtask $\theta_{m}^{s}$, where $t_{r}$ is the end of duration that spans from $t_{1}$ toward $t_{2}$, $\forall i\neq i^{\prime }\in N$.

(1) $$EL_{ink}^{i,i^{\prime }}\left(\theta_{m}^{s},t_{2}\right)=EL_{ink}^{i,i^{\prime \prime }}\left(\theta_{m}^{s},t_{1}\right)+\sum_{r\in \Delta t}I_{i}^{i^{\prime }}\left(\theta_{m}^{s},t_{r}\right)$$

Implicit links, i.e., $IL_{ink}$, are traditionally observed through triadic closure theory [34]. Triadic closure, in short, asserts that for each three agents *i*, $i^{\prime }$ and $i^{\prime \prime }$ where two explicit affinities exist in terms that link $i↔i^{\prime }$ and $i^{\prime }↔i^{\prime \prime }$, there should exist an implicit affinity that links $i↔i^{\prime \prime }$. In a triadic formation of two explicit affinities, there are different possibilities for the value ∈R that the implicit affinity should have. The possible value that an implicit affinity may obtain depends on the value of the current explicit edges. Thus, we can state that the initial value of the third implicit link, i.e., $IL_{ink}^{i,i^{\prime \prime }}$, in a triad can be approximated in Equation (2), which is $\forall i\neq i^{\prime }\neq i^{\prime \prime }\in N$.

(2) $$IL_{ink}^{i,i^{\prime \prime }}\left(\theta_{m}^{s}\right)\equiv \frac{EL_{ink}^{i,i^{\prime }}\left(\theta_{m}^{s}\right)+EL_{ink}^{i^{\prime },i^{\prime \prime }}\left(\theta_{m}^{s}\right)}{|R_{elation}^{i,i^{\prime }}\left(\theta_{m}\right)+R_{elation}^{i^{\prime },i^{\prime \prime }}\left(\theta_{m}\right){|}^{2}}$$

We are considering the formation of implicit links through explicit links only. That means there must be an explicit path from the source node to target node in order for an implicit link to exist. The traversal in the path of unrepeated explicit links between *i* and $i^{\prime \prime }$ will consider the maximum volume despite distances. An extension of the closure envisioned in Equation (2), where there existed two disjoint, i.e., nonconsecutive, links with explicit affinities or possible undefined links in between, is determined through Equation (3).

(3) $$IL_{ink}^{i,i^{\prime }}\left(\theta_{m}^{s}\right)\equiv \frac{\sum_{i,i^{\prime }}EL_{ink}^{i,i^{\prime }}\left(\theta_{m}^{s}\right)}{|\sum_{i,i^{\prime }}R_{elation}^{i,i^{\prime }}\left(\theta_{m}\right){|}^{2}}$$

Agents’ interaction are instrumental in forming new implicit links and updating the values of existing explicit ones. During a task completion, it is possible for frequently used implicit relations to gain a sense of actualization; thereby, the implicit relations will be treated the same as explicit ones. Next, we model relations considering those measurements of explicit as well as implicit links. As stated earlier, the initial values of relations are provided by the agents’ public profiles and are used in forming a task-based socio-graph. We mapped those relations into explicit links in a task-based graph in order to capture current interaction as well as to allow possible measures of implicit links. By the time a new task is going to be assigned, an organization updates agents’ relations over all tasks based on the new values of links. When a relation from an implicit link ($IL_{ink}$) reaches a threshold value of τ that has been specified previously by an organization, it will be treated as an explicit one and an agent is able to explicitly form a relation over it. It is possible for those relations to have a value of positive, negative, or mutual (i.e., equal) for non-existing or possible unprejudiced relations.

The relations in the graph are asymmetric relations, so we have to know the temporal direction of those relations. That is, in a tuple $\langle i,EL_{ink},i^{\prime }\rangle$ we have to know if the relation direction is from *i* to $i^{\prime }$, i.e., $i\to i^{\prime }$, or from $i^{\prime }$ to *i*, i.e., $i\to i^{\prime }$. Equation (4) updates the initial value of relation between every pair of agents by considering the most repeated value over an explicit or an implicit link at a given subtask, that is $\forall \;i\neq i^{\prime }\in N$ and $\forall \;\theta_{m}^{s}\in \left\{\Theta \right\}$.

(4) $$R_{elation}^{i\to i^{\prime }}\left(\theta_{m}\right)=\underset{s}{\mathrm{mode}}\;\left(EL_{ink}^{i,i^{\prime }}\left(\theta_{m}^{s}\right)+IL_{ink}^{i,i^{\prime }}\left(\theta_{m}^{s}\right)\right)$$

#### 2.1.2. Capacity Measure

Agent’s capacity can be described as the absolute ability to accomplish tasks given the time constrains and interests. A measurement of an agent’s capacity is a critical issue and should be addressed once an agent joins an organization. This will eliminate the possibility of agent’s ineligibility to accomplish tasks when allocated to it. The value of capacity is dynamic and rapidly changing from one task to another. For simplicity, we consider capacity to be a combination of an agent’s innate (1) capabilities for the ability to achieve different tasks, extemporaneous (2) willingness to perform certain actions based on her preferences, and ad-lib (3) availability for her readiness to participate. Agents’ capabilities and willingnesses are provided in their public profiles while availabilities are ranging from [0→1] based on the task they occupy. Willingness is the degree of commitment to which an agent is ready to work hard to achieve the organizational objectives. The willingness of an agent is important in determining her contributions for a task. Equation (5) shows a very direct measurement for agent *i*’s capacity to achieve a certain task $\theta_{m}\in \left\{\Theta \right\}$ and that ∀i∈N.

(5) $$\begin{array}{c} C_{apacity}^{i}\left(\theta_{m}\right)={\mathrm{availability}}_{i}\left(\theta_{m}\right)\cdot \left[{\mathrm{capability}}_{i}\left(\theta_{m}\right)+{\mathrm{willingness}}_{i}\left(\theta_{m}\right)\right] \end{array}$$

Due to the rapid changes in the agent’s capacity, an agent will not be able to preserve them for future use. They must be updated instantaneously every time a new task is performed. We assume that the capacity of an agent is independent ∀i∈N. Along with the presentation of a possible capacity measure, we have proposed a measurement for an agent relations driven from their continual interactions. Those two main parameters needed in determining the values of agents’ unconditional contributions, which are going to be used in the following sections to help in defining measurements of benevolence.

#### 2.1.3. The Value of Benevolence

Agents entering an organization and interacting with those whom they have no previous interaction are initializing their benevolent values with a constant of a *Null*; then, the benevolences are derived from their relationships with others as well as their capacities to overcome certain problems. Due to the fact that an organization is a formation that overlays a dynamic network, we model benevolence between agents based on a directed network’s graph of connected vertices and edges. The resultant graph will be a task-based weighted graph of vertices as agents capacities and edges as their relations. The weighted benevolent graph is connected, and there should at least be one active relation between any pair of agents. We follow next with a formal definition of the weighted benevolent graph while emphasizing on the parameters that contribute to its value.

Let N⊆N be a set of agents working on a goal $G_{i}$. There exists a set of tasks, i.e., $\left\{\Theta \right\}=\langle \theta_{1},\;\cdots ,\;\theta_{m}\rangle$, for each goal. Let $w:2^{N}\to x$, where w(N)∈N is a world of *N*-agents working on $\theta_{m}$, and *x* is a random variable with distribution that has not been determined yet. The parameters of the w(N) are attained from an organization and sampled over existing *k*-subtasks to all *N*. Let $B_{enevolence}^{i}:R^{k}\to R$ be the benevolent function of real values that computes the benevolent value of w(N) at $\theta_{m}$ based on the distribution of *k*-sub-tasks. We are trying to find out the benevolent values resulting from unilateral relationships between agents of N⊆N in the w(N).

A benevolent socio-graph is basically a combination of agents and relations. The value of relations can be different from one task to another; however, for the sake of simplicity, we will be evaluating those relations in a task-based graph. We use the normal distribution to correspond to the average values of agents benevolences with a peak and the variability with other agents in a symmetric spread, i.e., $C_{apacity}^{i}=\left(C_{apacity}^{i,1},\;\cdots ,\;C_{apacity}^{i,k}\right)$, where *k* is number of subtasks and $C_{apacity}$ is the agent’s capacity $\forall \;C_{apacity}^{i,k}∼C_{apacity}\left(\mu_{i,k},\sigma_{i,k}^{2}\right)$. The benevolence between a pair of agents $\left(i,i^{\prime }\right)$ can be presented in Equation (6).

(6) $$B_{enevolence}^{i\to \neg i}\left(\theta_{m}\right)=R_{elation}^{i\to \neg i}\left(\theta_{m}\right)\cdot C_{apacity}^{i}\left(\theta_{m}\right)$$

The values of relations are critical in this case, they are resulting from a weighted directed graph of the network. The benevolence takes advantage of agents’ current relations and the rapid changes in their values within the assignment of one task. We take into consideration an agent current interests and readiness to contribute captured in the measurement of capacity. Although implicit links are not considered when defining benevolence, current values of relations have already considered them, and they will directly contribute to current values of benevolence once a specific threshold is reached.

#### 2.1.4. The Value of Potential-Benevolence

Agents’ beliefs play an important role in the expected receipt of SC. When an agent believes that another is able to provide resources to her, she will then try to obtain those resources. When resources are obtained, trust is initiated. Agents providing resources are then of higher power and importance than the agent acquiring them. Since the value of the SC that initializes the link from acquirer to provider is proportional to the acquirer belief, we consider belief to be a function of the directed link to the provider. The value obtained from this function is proportional to the value of the SC gained by the provider. Given a graph of *N*-nodes and *i* is one of the nodes while ¬i are other member nodes ∈N that are ≠i, the potential benevolence of agent *i* receiving a contribution from other agents within *N* is obtained through Equation (7).

(7) $$\begin{array}{c} PB_{enevolence}^{i}\left(\theta_{m}\right)=\sum_{\forall \neg i\in N}B_{elief}^{\neg i}\left(C_{apacity}^{i}\left(\theta_{m}\right)\cdot R_{elation}^{\neg i\to i}\left(\theta_{m}\right)\right) \end{array}$$

Equation (7) states that the value of an agent’s capacity is a critical parameter for receiving a benevolence. The value of a relation from i→¬i is not the sum of all links an agent traverses through to get to the provider. It can be calculated through an implicit link $IL_{ink}$ if an explicit directed link, i.e., $EL_{ink}$, is not available.

### 2.2. Measurement of SC

The SC for an agent is based on her beliefs of receiving contribution from peers over the network. The probability of an agent providing a continual benevolence to another is proportional to the expected capacity that the acquirer may be interested in, as stated in Equation (8). We are considering, in our measurements of SC, a task-based graph for which the following equations are for a specific task, e.g., $\theta_{m}$.

(8) $$f\left(B_{enevolence}^{\neg i\to i}|PB_{enevolence}^{\neg i\to i}\right)=\left\{\begin{array}{cc} \sum_{\forall \neg i\in N}\left[\frac{B_{enevolence}^{\neg i\to i}\cap PB_{enevolence}^{\neg i\to i}}{PB_{enevolence}^{\neg i\to i}}\right] & \;\mathrm{if}\;PB_{enevolence}>\;0\\ 0 & \;\;\mathrm{Otherwise}; \end{array}\right.$$

The intersection represents an expectation to receive benevolence considering the given benevolence; otherwise, the value of "zero" is considered. In our case, we can eliminate the value of potential benevolence after the intersection and assume that the value of benevolence is true if the potential one exists. Equation (9) shows that directed SC is gained by the provider agent.

(9) $$SC_{i}=\sum_{\forall \neg i\in N}B_{elief}^{i}\left(\;f\left(B_{enevolence}^{\neg i\to i}|PB_{enevolence}^{\neg i\to i}\right)\;\right)$$

We consider belief to be a decay function that decreases the value of SC received when traversing through multiple agents. It is exponential to how many explicit links the acquirer has to travel through to obtain resources from the agent provider. We introduce the belief function $B_{elief}:R^{+}\to R^{+}$, where $B_{elief}^{i\to \neg i}\left(R_{elation}^{i\to \neg i}\right)$ is the belief of the relation that returns the task based between agents *i* and ¬i. Belief is a monotonically decreasing function so that a larger number of relations corresponds to a lower belief. The belief value is domain specific and an example of it can be: $B_{elief}^{i}=e^{-\lambda \cdot (R_{elation}^{i})}$. When an agent capitalizes on another, her current capacity is also accessible for that agent to take advantage of, in-return. When both agents capitalize on each other, they form a cooperative behavior that contributes positively to the organizational SC, feeding back to the organization member-agents.

## 3. The Case of an OSS Project

Social phenomena, such as a lack of engagement, cohesion, or even corruption, have not always been observed instantly. Empirical researchers have traditionally been looking for pools to obtain information on perceptions instead. This is because of the natural contextual dependency in various abstract aspects of SC that only make sense in a unified context, which makes it difficult to come up with standardized identical measures. We, therefore, consider OSS to define our bounded organization and to ease the process of measuring different social aspects of SC that were challenging to compute efficiently in traditional methods.

Open source software is a type of software projects with publicly released source code and the users, in most cases, have the right to change the source code of the system. The development process of OSS projects are different than industrial software projects. OSS developments are based on collaborations between multiple independent developers, i.e., contributors, who aim to achieve a common goal. The contributors are usually located in different geographical areas. Thus, OSS projects mostly have online repositories, e.g., GitHub, that allow multiple developers to contribute independently to the project [4]. Over the last two decades, OSS development has gained popularity, and we have witnessed successful OSS projects such as Linux, MySQL, and Hadoop. However, the majority of OSS projects have failed due to different reasons, e.g., [35,36]. In this paper, we try to understand the impact of SC on OSS development and whether it has a relation with the success of OSS projects.

In order to find a relevant OSS project that is best fitted to the SC concept, we have taken different aspects into account, for example, interactions, implicit/explicit links, relationships, and capacities. Finally, we chose the Apache Software project as our research context, which has served as an example for OSS in many previous studies, e.g., [37]. The Apache Hadoop project was initialized by Apache Software Foundation in 2003 and has been one of the most active OSS projects since its beginning. There are software releases and evolution of the software since then, which are possible because of the fast-moving development process and the broad foundation of contributors, ranging from hobbyists to companies. Thus, it involves more people than other OSS projects.

### 3.1. Determining Parameters

We consider the OSS Project Hadoop as a case study to illustrate the SC value computation. We focus on org.apache.hadoop.yarn.client.api package that has 10 classes and 31 contributors involved in the package development. *Apache Hadoop YARN* is the resource management and job scheduling technology in the open source Hadoop distributed processing framework. YARN is responsible for allocating system resources to the various applications running in a Hadoop cluster and scheduling tasks to be executed on different cluster nodes. The technology became an Apache Hadoop subproject within the *Apache Software Foundation* (ASF) in 2012 and was one of the key features added in *Hadoop 2.0*, which was released for testing that year and became generally available in October 2013. The data are taken from the GitHub portal from year 2013 to 2018, and we divide the data into three time intervals ($t_{1}$: 2013–2014, $t_{2}$: 2015–2016, and $t_{3}$: 2017–2018). In this case study, the task is the package org.apache.hadoop.yarn.client.api in the project, and the subtasks are the 10 classes in the package, i.e., $\theta_{m}^{\left\{10\right\}}$. We consider the number of line code as the value of interaction among the contributors, i.e., adding and deleting lines.

Table 1 shows the names of the 10 sub-tasks (classes), number of commits, and the number of agents involved in each time interval. Class number 2 and Class number 8 involves more than 4 agents, for each time interval, that constitute social networks, while Class number 5 involve more than 1 agent in time interval $t_{2}$ and $t_{3}$. The rest of the classes have only one agent involved, thus, they do not constitute a social network, thus do not contribute any social capital value. We give an example of a social capital computation of agents involved in Class number 2 for time interval $t_{1}$ to illustrate the overall social capital computation of this social organization network.

**Figure 1:**
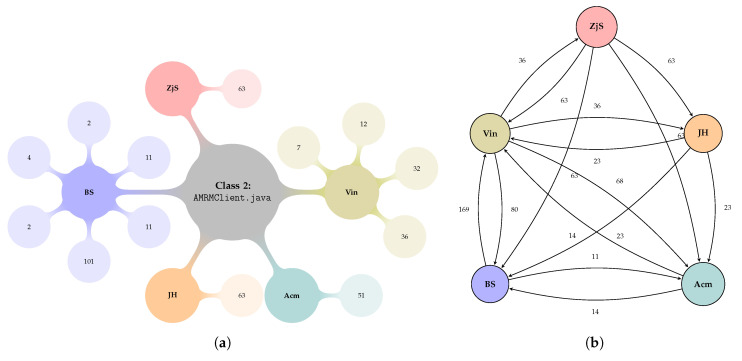
Illustrative sample of the social network organization constituted by contributors in class AMRMClient.java for time interval 2013–2018. (**a**) Mapping the contributions of every agent; (**b**) Ascending order of contributions.

Table 2 shows sample data that has been collected from the GitHub portal for AMRMClient.java class of org.apache.hadoop.yarn.client.api package. The class involves 16 contributors throughout the development period with the interval of: 2013–2018. The sample data illustrated in Table 2 constitutes a network organization based on the social fabric of contributions. A smaller social formation of the AMRMClient.java class at the time interval $t_{1}$: 2013–2014 has been constructed by 5 members with various contributions to the class. Figure 1 shows a graph-based agents contributions to the class in focus—Class 2. Therefore, the aggregation of all social formations constructed to solve a given problem, i.e., when the agents are working on specific classes, for the 10 classes within the package forms the overall network of this package as an organization.

A social network of contributors is constituted based on the data presented in Table 2. As an illustrative example, the social network of AMRMClient.java class for time interval ($t_{1}$: 2013-2014) is constructed by 5 members contribute to the class (Figure 1a) and create a social network as shown in Figure 1b. Thus, the overall social network of this package is a union of social networks of the 10 classes in the package.

Table 3 shows the total involvement of every agent within the organization and their relations to one another that have been calculated from all 10 classes of the package. Also, the number of classes, in which each contributor was involved, has been counted in order to later help in the measurement of capacity for every individual agent. Moreover, due to the openness nature of an organization such as this OOS project, we based our assumption on the premise that the willingness of the contributors is at a maximum level, i.e., the value of willingness = 1. Once the data input processing is complete, the SC of every individual agent can be computed using the Algorithms 1 and 2 proposed.

**Algorithm 1:** A Sociograph based on Relations.

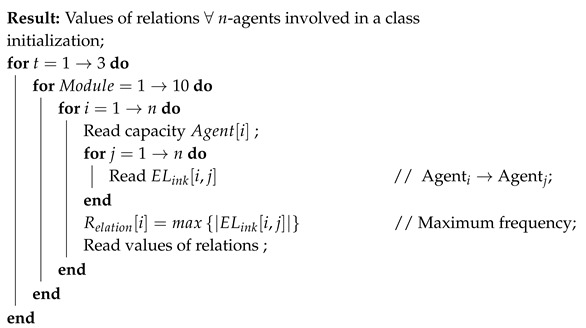



**Algorithm 2:** Measurements of Social Capital.

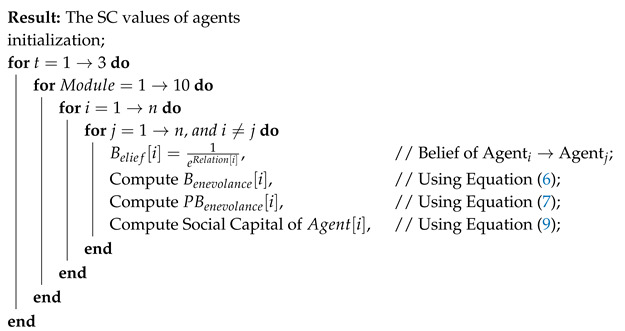



### 3.2. An Illustrative Example: Class 2

To illustrate the overall computation of the social capital of this social formation, this section presents a calculation of SC with a sample collection of data from a sub-task-2 (Class-2) throughout the time interval $t_{1}$: 2013–2014. Table 4 depicts the data used in the social capital calculation of the class. The relation values are the total number of contributions, i.e., the total code lines provided to include addition as well as deletion.

#### 3.2.1. Links

**Figure 2:**
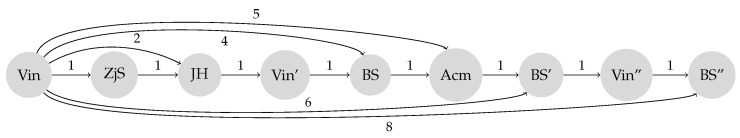
The value of relation of each node in the social network in order to measure the belief values. Here, we consider the repetition of agents’ order in the network when showing up again with apostrophes.

Based on the assumption that an agent benevolence to one class is equal to every individual member within that class, the number of agents involved within this class have been sorted in ascending order considering their time of contribution. Figure 2 illustrates the number of links in the network to calculate the belief values.

We are now able to calculate the link for each contributor by implementing Algorithms 1 and 2. The links for contributor *Vin*, for instance, are: $EL_{ink}\left(Vin,ZjS\right)=1$; $EL_{ink}\left(Vin,JH\right)=2$; $EL_{ink}\left(Vin,BS\right)=\left\{4,6,8\right\}$; $EL_{ink}\left(Vin,Acm\right)=5$. By the use of Equation (4), we are able to obtain the value of relations for all contributors, as shown in Table 5.

#### 3.2.2. Relation

Table 6 shows the values of relations among the contributors in the Class-2 network. The value of an agent relation is the sum-up number of code lines to include addition and deletion from each contributor (refer to Table 3).

#### 3.2.3. Belief

Belief: $B_{elief}^{i}=e^{-\lambda \cdot (R_{elation}^{i})}$. Assume that λ=1. Table 7 shows the Belief values from one contributor relative to the others.

#### 3.2.4. Benevolence

Benevolence is calculated as defined in Equation (6). Table 8 shows the calculation results.

#### 3.2.5. Potential Benevolence

The value of Potential Benevolences are calculated using Equation (7). The values of Relations are obtained from Table 3 (sum up the addition and deletion), and the values of Belief are obtained from Table 5. The results are as follows.

$$\begin{array}{cc} PB_{enevolence}^{Vin} & =\left\{\left(B_{elief}^{ZjS}\times R_{elation}^{ZjS\to Vin}\right)+\left(B_{elief}^{JH}\times R_{elation}^{JH\to Vin}\right)+\left(B_{elief}^{BS}\times R_{elation}^{BS\to Vin}\right)+\left(B_{elief}^{Acm}\times R_{elation}^{Acm\to Vin}\right)\right\}\times C_{apacity}^{Vin}\\ & =\left\{(0.367\times 63)+(0.135\times 46)+(0.049\times 69)+(0.006\times 14)\right\}\times 6\\ & =22.176 \end{array}$$

$$\begin{array}{cc} PB_{enevolence}^{ZjS} & =\left\{\left(B_{elief}^{Vin}\times R_{elation}^{Vin\to ZjS}\right)+\left(B_{elief}^{JH}\times R_{elation}^{JH\to ZjS}\right)+\left(B_{elief}^{BS}\times R_{elation}^{BS\to ZjS}\right)+\left(B_{elief}^{Acm}\times R_{elation}^{Acm\to ZjS}\right)\right\}\times C_{apacity}^{ZjS}\\ & =\left\{(0.0003\times 36)+(0.135\times 46)+(0.0009\times 0)+(0.018\times 0)\right\}\times 1=8.452 \end{array}$$

$$\begin{array}{cc} PB_{enevolence}^{JH} & =\left\{\left(B_{elief}^{Vin}\times R_{elation}^{Vin\to JH}\right)+\left(B_{elief}^{ZjS}\times R_{elation}^{ZjS\to JH}\right)+\left(B_{elief}^{BS}\times R_{elation}^{BS\to JH}\right)+\left(B_{elief}^{Acm}\times R_{elation}^{Acm\to JH}\right)\right\}\times C_{apacity}^{JH}\\ & =\left\{(0.0009\times 36)+(0\times 63)+(0.0009\times 0)+(0\times 0)\right\}\times 3=0.0972 \end{array}$$

$$\begin{array}{cc} PB_{enevolence}^{BS} & =\left\{\left(B_{elief}^{Vin}\times R_{elation}^{Vin\to BS}\right)+\left(B_{elief}^{JH}\times R_{elation}^{JH\to BS}\right)+\left(B_{elief}^{JH}\times R_{elation}^{JH\to ZjS}\right)+\left(B_{elief}^{Acm}\times R_{elation}^{Acm\to BS}\right)\right\}\times C_{apacity}^{BS}\\ & =\left\{(0.018\times 169)+0+0+(0.367\times 14)\right\}\times 3=24.54 \end{array}$$

$$\begin{array}{cc} PB_{enevolence}^{Acm} & =\left\{\left(B_{elief}^{Vin}\times R_{elation}^{Vin\to Acm}\right)+\left(B_{elief}^{JH}\times R_{elation}^{JH\to Acm}\right)+\left(B_{elief}^{BS}\times R_{elation}^{BS\to Acm}\right)+\left(B_{elief}^{BS}\times R_{elation}^{BS\to Acm}\right)\right\}\times C_{apacity}^{Acm}\\ & =\left\{(0.018\times 14)+0+0+(0.049\times 11)\right\}\times 3=2.373 \end{array}$$

#### 3.2.6. Social Capital

According to Equation (9), the value of SCs are calculated as follows:

$$\begin{array}{cc} SC^{Vin}= & \left\{B_{elief}^{Vin\to ZjS}\times \left[\frac{\left(B_{enevolence}^{ZjS\to Vin}\cap PB_{enevolence}^{ZjS\to Vin}\right)}{PB_{enevolence}^{ZjS\to Vin}}\right]\right\}+\left\{B_{elief}^{Vin\to JH}\times \left[\frac{\left(B_{enevolence}^{JH\to Vin}\cap PB_{enevolence}^{JH\to Vin}\right)}{PB_{enevolence}^{JH\to Vin}}\right]\right\}\\ & +\left\{B_{elief}^{Vin\to BS}\times \left[\frac{\left(B_{enevolence}^{BS\to Vin}\cap PB_{enevolence}^{BS\to Vin}\right)}{PB_{enevolence}^{BS\to Vin}}\right]\right\}+\left\{B_{elief}^{Vin\to Acm}\times \left[\frac{\left(B_{enevolence}^{Acm\to Vin}\cap PB_{enevolence}^{Acm\to Vin}\right)}{PB_{enevolence}^{Acm\to Vin}}\right]\right\}\\ = & \left\{0.367\times \frac{\left(63-8.452\right)}{8.452}\right\}+\left\{0.135\times \frac{\left(138-1.944\right)}{1.944}\right\}+\left\{0.049\times \frac{\left(1521-24.54\right)}{24.54}\right\}+\left\{0.006\times \frac{\left(126-2.373\right)}{2.373}\right\}\\ = & \;15.117 \end{array}$$

$$\begin{array}{cc} SC^{ZjS}= & \left\{B_{elief}^{ZjS\to Vin}\times \left[\frac{\left(B_{enevolence}^{Vin\to ZjS}\cap PB_{enevolence}^{Vin\to ZjS}\right)}{PB_{enevolence}^{Vin\to ZjS}}\right]\right\}+\left\{B_{elief}^{ZjS\to JH}\times \left[\frac{\left(B_{enevolence}^{JH\to ZjS}\cap PB_{enevolence}^{JH\to ZjS}\right)}{PB_{enevolence}^{JH\to ZjS}}\right]\right\}\\ & +\left\{B_{elief}^{ZjS\to BS}\times \left[\frac{\left(B_{enevolence}^{BS\to ZjS}\to PB_{enevolence}^{BS\to ZjS}\right)}{PB_{enevolence}^{BS\to ZjS}}\right]\right\}+\left\{B_{elief}^{ZjS\to Acm}\times \left[\frac{\left(B_{enevolence}^{Acm\to ZjS}\cap PB_{enevolence}^{Acm\to ZjS}\right)}{PB_{enevolence}^{Acm\to ZjS}}\right]\right\}\\ = & \left\{0.0003\times \frac{\left(63-226.176\right)}{226.176}+0.367\times \frac{\left(0-1.944\right)}{1.944}+0.0009\times \frac{\left(0-24.54\right)}{24.54}+0.018\times \frac{\left(0-2.373\right)}{2.373}\right\}\\ = & -0.386 \end{array}$$

$$\begin{array}{cc} SC^{JH}= & \left\{B_{elief}^{JH\to ZjS}\times \left[\frac{\left(B_{enevolence}^{ZjS\to JH}\cap PB_{enevolence}^{ZjS\to JH}\right)}{PB_{enevolence}^{ZjS\to JH}}\right]\right\}+\left\{B_{elief}^{JH\to Vin}\times \left[\frac{\left(B_{enevolence}^{Vin\to JH}\cap PB_{enevolence}^{Vin\to JH}\right)}{PB_{enevolence}^{Vin\to JH}}\right]\right\}\\ & +\left\{B_{elief}^{JH\to BS}\times \left[\frac{\left(B_{enevolence}^{BS\to JH}\cap PB_{enevolence}^{BS\to JH}\right)}{PB_{enevolence}^{BS\to JH}}\right]\right\}+\left\{B_{elief}^{JH\to Acm}\times \left[\frac{\left(B_{enevolence}^{Acm\to JH}\cap PB_{enevolence}^{Acm\to JH}\right)}{PB_{enevolence}^{Acm\to JH}}\right]\right\}\\ = & \left\{0.0009\times \frac{\left(1296-226.176\right)}{226.176}+0.0\times \frac{\left(63-8.452\right)}{8.452}+0.0009\times \frac{\left(0-24.54\right)}{24.54}+0.018\times \frac{\left(0-2.373\right)}{2.373}\right\}\\ = & -0.014 \end{array}$$

$$\begin{array}{cc} SC^{BS}= & \left\{B_{elief}^{BS\to ZjS}\times \left[\frac{\left(B_{enevolence}^{ZjS\to BS}\cap PB_{enevolence}^{ZjS\to BS}\right)}{PB_{enevolence}^{ZjS\to BS}}\right]\right\}+\left\{B_{elief}^{BS\to JH}\times \left[\frac{\left(B_{enevolence}^{JH\to BS}\cap PB_{enevolence}^{JH\to BS}\right)}{PB_{enevolence}^{JH\to BS}}\right]\right\}\\ & +\left\{B_{elief}^{BS\to Vin}\times \left[\frac{\left(B_{enevolence}^{Vin\to BS}\cap PB_{enevolence}^{Vin\to BS}\right)}{PB_{enevolence}^{Vin\to BS}}\right]\right\}+\left\{B_{elief}^{BS\to Acm}\times \left[\frac{\left(B_{enevolence}^{Acm\to BS}\cap PB_{enevolence}^{Acm\to BS}\right)}{PB_{enevolence}^{Acm\to BS}}\right]\right\}\\ = & \left\{0.018\times \frac{\left(2880-226.176\right)}{226.176}+0.0\times \frac{\left(68-8.452\right)}{8.452}+0.0\times \frac{\left(138-1.944\right)}{1.944}+0.367\times \frac{\left(126-2.373\right)}{2.373}\right\}\\ = & \;19.33 \end{array}$$

$$\begin{array}{cc} SC^{Acm}= & \left\{B_{elief}^{Acm\to ZjS}\times \left[\frac{\left(B_{enevolence}^{ZjS\to Acm}\cap PB_{enevolence}^{ZjS\to Acm}\right)}{PB_{enevolence}^{ZjS\to Acm}}\right]\right\}+\left\{B_{elief}^{Acm\to JH}\times \left[\frac{\left(B_{enevolence}^{JH\to Acm}\cap PB_{enevolence}^{JH\to Acm}\right)}{PB_{enevolence}^{JH\to Acm}}\right]\right\}\\ & +\left\{B_{elief}^{Acm\to BS}\times \left[\frac{\left(B_{enevolence}^{BS\to Acm}\cap PB_{enevolence}^{BS\to Acm}\right)}{PB_{enevolence}^{BS\to Acm}}\right]\right\}+\left\{B_{elief}^{Acm\to Vin}\times \left[\frac{\left(B_{enevolence}^{Vin\to Acm}\cap PB_{enevolence}^{Vin\to Acm}\right)}{PB_{enevolence}^{Vin\to Acm}}\right]\right\}\\ = & \left\{0.018\times \frac{\left(2880-226.176\right)}{226.176}+0.0\times \frac{\left(63-8.452\right)}{8.452}+0.0\times \frac{\left(138-1.944\right)}{1.944}+0.049\times \frac{\left(99-24.54\right)}{24.54}\right\}\\ = & \;0.359 \end{array}$$

Table 9 shows the completed computation of SCs of Class number 2 for the three time intervals. The SCs values are accumulated from previous time intervals.

#### 3.2.7. Measuring SC for Only One Agent in the Subtask

As shown in Table 1, in each time interval there are some sub-tasks (classes) that consist of only one contributor. Since there is only one agent/contributor in the class, we assume the relation is 1 (self relation), then the Belief is $\frac{1}{e}$ and $PB_{enevolence}=0$. Hence, $PB_{enevolence}=0$, then, according to Equation (8), the value of $f(B_{enevolence}|PB_{enevolence})=0$, thus the value of SC is zero. The calculation of the SC for this case is shown in Table 10.

#### 3.2.8. Overall Social Capital

As mentioned in Section 3.1, the overall value of social capital is computed using the Algorithms 1 and 2. Table 11 shows the overall SCs of each contributor for intervals $t_{1}$, $t_{2}$ and $t_{2}$. The social capital value of each agent/contributor is a sum up of contributions in the involved sub-tasks/classes.

### 3.3. Discussion

Here, we discuss social capital from four different perspectives as observed in the previous stated case study.

1. From the point of *Relations*, as mentioned in Section 2.1.1, the existing relations among the agents are explicit links among them and the calculation of relations is illustrated in Figure 2 and Table 5. As we can see from Figure 2, relations/links are constructed based on the agent’s commit dates. The more agents involved, the more commit dates, thus, the more links created. Then, a class with only two contributors/agents implies that the agents have only 1 link and gives a high belief value. A sole agent in a class does not develop any social capital since it has no relations. Her contribution will be taken into consideration when we consider the relation among classes, which is based on inherited methods from each others classes reflecting inter-organizational perspective is, however, out of the scope of the experiment.
2. From the *Beliefs* perspective, we recall from Section 2.2 that Belief is a monotonically decreasing function so that a larger number of relations corresponds to a lower belief. In the case of the OSS organization, each contributor does not know each other; thus, we merely measure the belief based on the number of links corresponding to the agents. So, it makes sense that in a class with only two agents/contributors, the agent trusts each other.
3. From the view of *Benevolences*, in the observed Package, there are three classes that involve more than 2 contributors: Class 2 (AMRMClient.java) with 16 contributors, Class 5 (NMClient.java) with 6 contributors, and Class 8 (YarnClient.java) with 21 contributors. Due to the nature of the OSS organization that contributors/agents are not bonding (volunteer based involvement), we have observed that only one agent (JH) consistently contributes in the three time intervals. The results have shown that some individual agents, at the end of each time interval or even the complete project, ended up with negative values of SC, which is because of the higher expectations to receive benevolences from their peers. This means current connections and the promising values they may entail may not always be available to share, which is, when considering the benevolent behavior, what this paper is about. Nevertheless, the overall values of SC for each time interval are positive. The SC value increases 814.97% and 102.36% for time interval $t_{2}$ and time interval $t_{3}$, respectively. The value of SC during time interval $t_{2}$ has peaked due to the high benevolence a single agent has contributed to the network. During this time interval, two agents—including agent Oz—are involved in class number 5. Since only two agents are involved, the relation among the agents is 1, giving the value of $B_{elief}$ to be close to 1; therefore, agent Oz contributes a higher benevolence value (Table 11). This, in part, indicates that an agent productivity contributes positively to the organizational outcome.
4. Form the *Organizational goal*, the publicly available data of the observed package of Apache Hadoop can be summarized as the number of commit dates of agents to complete designated tasks for each time interval, $t_{1}$, $t_{2}$, and $t_{3}$, are 37, 37, and 28, respectively. We conclude, when we consider the number of commits, that the agents in this organizational network have successfully delivered the project and, accordingly, they have positive SC values that have led to a positive value of their organization.

## 4. Conclusions and Future Work

The idea of this paper is to reflect the effects of existing social fabric among individual agents by quantifying “Social Capital” in an ad-hoc organization using benevolence as a measure of their collaborations. We illustrated that SC is achievable in the context of a dynamic network organization with the help of a popular open source software project through the presentation of heuristics to compute numeric values for measuring SC, which, in turn, can translate to degree and effectiveness of collaboration in open ad-hoc agent organizations. This article defined SC on three different levels, i.e., network, link and agent, and proposed a measurement for it based on the benevolences between autonomous agents operating in a large-scale open service-oriented organization. Incorporating benevolence, in measuring the social capital for individual agent and for the organization as whole, gives more tangible values. Those values contribute positively towards the cooperative nature of an organization. We showed an empirical evaluation of the proposed approach using a real-world case study of an open-source project development, and we assessed the validity of each measure of SC in different settings within a network organization. Furthermore, the empirical evaluation showed that the social capital of an individual agent may result in a negative value as the agent expected contributions from other agents; however, the contribution received may not be as expected. This finding is a result of considering the benevolence in a social capital measurement. Another finding is that the belief and trust also contributes towards the measurement of social capital. As we observe, the social capital of a group or an organization increases when it involves more agents; on the other hand, few numbers of agents involved in a subtask with significant line code contributions provides a higher social capital, due to a higher value of belief. The authors believe that the amount of data used in the empirical evaluation represents the behavior of the OSS social network, as the computations are to be easily scaled up.

The concept of SC has shown its usefulness, fruitfulness, and efficiency in genuine empirical research, such as the available empirical approach of SC presented here. The heterogeneity of the proposed conceptualization has been less reflected in the empirical heterogeneity as to what has been expected. Future work should consider the use of multi-method and multi-level strategies to improve the current role of empirical evidence in the debate on SC. It should also consider the proposition of a detailed social capital assessment model that is required to estimate the future behavior of agents and agents’ peers in order to simplify the interaction process with those peers. Another further direction is to benefit from a systematic analysis and recommendations on how agents ought to behave for better performance to include comparisons with baselines and other metrics that measure ad-hoc organizations.

## Figures and Tables

**Table 1:** Classes and contributors of the package: org.apache.hadoop.yarn.client.api.

No.	Class Name	# of Commits	Involved Agents			Total
			$t_{1}$	$t_{2}$	$t_{3}$	
1	AHSClient.java	3	0	2	1	3
2	AMRMClient.java	37	5	8	9	16
3	ContainerShellWebSocket.java	2	0	0	1	1
4	InvalidContainerRequestException.java	1	1	0	0	1
5	NMClient.java	9	1	2	3	6
6	NMTokenCache.java	4	1	1	1	3
7	SharedCacheClient.java	3	0	1	2	3
8	YarnClient.java	42	7	12	5	21
9	YarnClientApplication.java	1	1	0	0	1
10	Package-info.java	1	1	0	0	1

**Table 2:** Class 2: AMRMClient.java, # of contributors =16.

Commit Date	Contributor	Add #	Del#	Commit Date	Contributor	Add #	Del #
18 June 2013	Vinoduec (Vin)	4	3	26 April 2016	Xslogic (Xs)	2	1
10 June 2013	Bikas Saha - (BS)	1	1	12 June 2016	Xslogic (Xs)	35	8
21 June 2013	Vinoduec (Vin)	0	12	10 July 2016	Sjlee (SjL)	5	5
15 July 2013	Bikas Saha (BS)	10	1	09 August 2016	Rohithsharmaks (Roh)	1	1
16 July 2013 (1)	Bikas Saha (BS)	85	16	27 August 2016	Wangdatan (WDT)	169	31
16 July 2013 (2)	Bikas Saha (BS)	1	1	12 November 2016	Wangdatan (WDT)	88	21
17 July 2013	Bikas Saha (BS)	6	41	15 November 2016	Xslogic (Xs)	116	1
19 July 2013	Acmurthy - (Acm)	5	9	14 February 2017	Xslogic (Xs)	31	2
31 August 2013	Bikas Saha (BS)	11	0	14 February 2017	Xslogic (Xs)	31	2
31 October 2013	Vinoduec (Vin)	32	0	16 February 2017	Sjlee (SjL)	30	10
09 July 2014	jian-he - (JH)	15	8	30 August 2017	jian-he (JH)	3	2
09 August 2014	zjshen14 - (ZjS)	63	0	04 September 2017	Varunsaxena (Var)	20	2
11 October 2014	Vinoduec (Vin)	34	2	09 September 2017	Haibchen (Hai)	4	4
06 February 2015	jian-he (JH)	2	1	18 September 2017	Xslogic (Xs)	7	2
16 March 2015	Oza - (Oz)	2	2	31 October 2017	Aajisaka (Aaj)	4	3
18 March 2015	zjshen14 (ZjS)	18	0	17 January 2018	wangdatan (WDT)	37	1
19 March 2015	JunpingDu - (JPD)	3	3	16 February 2018	Sunilgovind (Sun)	12	65
12 November 2015	Wangdatan - (WDT)	23	2	31 July 2018	Hungj (Hun)	11	0
08 January 2016	Xslogic - (Xs)	40	3				

**Table 3:** Relations of contributors and their involvement.

No.	Contributor	# of Relations			# Classes	No.	Contributor	# of Relations			# Classes
		$t_{1}$	$t_{2}$	$t_{3}$				$t_{1}$	$t_{2}$	$t_{3}$	
1	Vin	24	18	1	6	17	Bil	0	0	3	2
2	Oz	0	26	0	5	18	Mac	0	0	2	1
3	Roh	0	21	0	3	19	Ctr	0	1	0	1
4	BS	12	0	0	3	20	Kam	0	0	1	1
5	Acm	13	0	0	3	21	Hit	7	0	0	1
6	JH	6	25	12	3	22	Ale	5	0	0	1
7	ZjS	6	7	0	1	23	Sry	6	0	0	1
8	JPD	0	7	0	1	24	Sub	6	0	0	1
9	WDT	0	27	13	4	25	Xgo	0	14	0	1
10	Xs	0	10	14	2	26	Jlo	0	14	0	1
11	SjL	0	9	11	2	27	Car	0	12	0	1
12	Var	0	0	8	1	28	Min	0	11	0	1
13	Hai	0	0	8	1	29	Nag	0	11	4	1
14	Aaj	0	12	5	3	30	Vas	0	10	0	1
15	Sun	0	12	7	2	31	Bib	0	0	4	1
16	Hun	0	0	7	1						

**Table 4:** Class name: AMRMClient.java, (2013-2014), # of contributors: 5.

Commit Data	Contributor/Agent	# Add	# Del	Relation	Capacity
18 June 2013	Vinoduec (Vin)	4	3	7	6
19 June 2013	Bikas Saha (BS)	1	1	2	3
21 June 2013	Vinoduec (Vin)	0	12	12	0
15 July 2013	Bikas Saha (BS)	10	1	11	0
16 July 2013 (1)	Bikas Saha (BS)	85	16	101	0
16 July 2013 (2)	Bikas Saha(BS)	1	1	2	0
17 July 2013	Bikas Saha (BS)	6	41	42	0
19 July 2013	Acmurthy (Acm)	5	9	14	3
31 August 2013	Bikas Saha (BS)	11	0	11	0
31 October 2013	Vinoduec (Vin)	32	0	32	0
09 July 2014	Jian-he (JH)	15	8	23	3
09 August 2014	Zjshen14 (ZjS)	63	0	63	1
11 October 2014	Vinoduec (Vin)	34	2	36	0

**Table 5:** Number of relations for all contributors in sub-task 2 (Class-2), i.e., $R_{elation}^{i\to \neg i}\forall i\neq \neg i\in N$.

	¬i	Vin	ZjS	JH	BS	Acm
*i*						
**Vin**		-	1	2	7	5
**ZjS**		8	-	1	7	4
**JH**		7	-	-	6	3
**BS**		4	-	-	-	1
**Acm**		4	-	-	3	-

**Table 6:** Values of relations among the contributors.

	Contributor	Vin	ZjS	JH	BS	Acm	Total
Receiver							
**Vin**		-	36	36	80	68	227
**ZjS**		63	-	63	63	63	252
**JH**		46	23	-	46	46	161
**BS**		169	-	-	-	11	180
**Acm**		14	-	-	14	-	28
**Total**		299	59	99	203	177	-

**Table 7:** Belief values, $B_{elief}^{i\to \neg i}$.

	*i*	Vin	ZjS	JH	BS	Acm
¬i						
**Vin**		-	0.367	0.135	0.049	0.006
**ZjS**		0.0003	-	0.367	0.0009	0.018
**JH**		0.0009	-	-	0.002	0.049
**BS**		0.018	-	-	-	0.367
**Acm**		0.018	-	-	0.049	-

**Table 8:** Benevolence for each contributor, i.e., $B_{enevolence}^{i\to \neg i}$.

	*i*	Vin	ZjS	JH	BS	Acm
¬i						
**Vin**		0	216 × 6 = 1296	216 × 6 = 1296	480 × 6 = 2880	408 × 6 = 2880
**ZjS**		63 × 1 = 63	0	63 × 1 = 63	63 × 1 = 63	63 × 1 = 63
**JH**		46 × 3 = 138	0	0	46 × 3 = 138	46 × 3 = 138
**BS**		507 × 3 = 1521	0	0	0	33 × 3 = 99
**Acm**		42 × 3 = 126	0	0	42 × 3 = 126	0

**Table 9:** Social Capitals of the agents involved in Class-2.

Agents	SC		
	$t_{1}$	$t_{2}$	$t_{3}$
**Vin**	15.117	15.117	15.117
**ZjS**	−0.386	3.17	3.17
**JH**	−0.014	4.773	10.171
**BS**	19.33	19.33	19.33
**Acm**	0.359	0.359	0.359
**Oza**	0	0.766	0.766
**JPD**	0	−1.023	−1.023
**WDT**	0	−0.986	1.126
**Xsl**	0	4.781	11.648
**SjL**	0	14.433	33.067
**Roh**	0	16.551	16.551
**Var**	0	0	−1.237
**Hai**	0	0	2.459
**Aaj**	0	0	7.889
**Sun**	0	0	1.252
**Hun**	0	0	0.746
**Total**	34.406	77.271	121.391

**Table 10:** SC of a single agent in a class.

Interval	Class #	Agent	$C_{apacity}$	$R_{elatin}$	$B_{enevelence}$	${PB}_{enevelence}$	SC
$t_{1}$	4	**BS**	3	38	114	0	0
	5	**Vin**	6	100	600	0	0
	6	**Vin**	6	143	858	0	0
	9	**Acm**	3	21	63	0	0
	10	**Vin**	6	52	312	0	0
$t_{2}$	6	**Oz**	5	58	290	0	0
	7	**Ctr**	1	108	108	0	0
$t_{3}$	1	**Vin**	6	10	60	0	0
	3	**Bil**	2	163	326	0	0
	6	**Aaj**	3	2	6	0	0

**Table 11:** The SCs for contributing agents.

Agent	SC			Agent	SC		
	$t_{1}$	$t_{2}$	$t_{3}$		$t_{1}$	$t_{2}$	$t_{3}$
**Vin**	35.558	79.461	79.461	**Aaj**	0	0.788	8.238
**BS**	22.21	22.21	22.21	**Min**	0	1.001	1.001
**Acm**	0.161	0.161	0.161	**Nag**	0	0.742	2.188
**JH**	−0.014	26.016	31.414	**Var**	0	0.689	−0.548
**ZjS**	−0.386	3.17	3.17	**Bib**	0	0	0.994
**Oz**	0	297.831	297.831	**Bil**	0	0	−0.897
**JPD**	0	−1.023	−1.023	**Hit**	1.112	1.112	1.112
**WDT**	0	18.909	22.386	**Ale**	0.887	0.887	0.887
**Xs**	0	4.781	13.192	**Sry**	1.086	1.086	1.086
**SjL**	0	14.433	33.067	**Sub**	−0.245	−0.245	−0.245
**Roh**	0	16.649	16.649	**Vas**	0	0	0
**Sun**	0	0.067	1.319	**Ctr**	0	0	0
**Hun**	0	0	0.746	**Kam**	0	0	−1.779
**Xgo**	0	1.347	1.347	**Hai**	0	0	2.459
**Jlo**	0	1.002	1.002	**Mac**	0	0	1.266
**Car**	0	0.92	0.92				
				**Total**	60.369	552.363	1091.977

## Abbreviations

The following abbreviations are used in this manuscript:

OOS	Open Source Software.
MAS	MultiAgent System.
SC	Social Capital and $SC^{i}$ is the social capital value of agent *i*.
$I_{i}^{i^{\prime }}$	Interaction between agent *i* and $i^{\prime }$.
$EL_{ink}^{i,i^{\prime }}$	Explicit link between agent *i* to $i^{\prime }$.
$IL_{ink}^{i,i^{\prime }}$	Implicit link between agent *i* and $i^{\prime }$.
$R_{elation}^{i\to i^{\prime }}$	Direct relationship from agent *i* to agent $i^{\prime }$.
$C_{apacity}^{i}$	The capacity that agent *i* has.
$B_{enevolence}^{i\to i^{\prime }}$	The directed benevolence from agent *i* and agent $i^{\prime }$.
$PB_{enevolence}^{i\to i^{\prime }}$	Directed potential benevolence agent $i^{\prime }$ expect to receive from agent *i*.
